# Supplementary material for: Regulation of the terminal maturation of iNKT cells by mediator complex subunit 23
Source: Nat Commun. 2018 Sep 24;9:3875. doi: 10.1038/s41467-018-06372-1 (PMC6155209; doi:10.1038/s41467-018-06372-1)
Supplement: Supplementary file 1 — Supplementary information [file 41467_2018_6372_MOESM1_ESM.pdf]

Regulation of the terminal maturation of iNKT cells by mediator complex subunit 23

Xu et al.

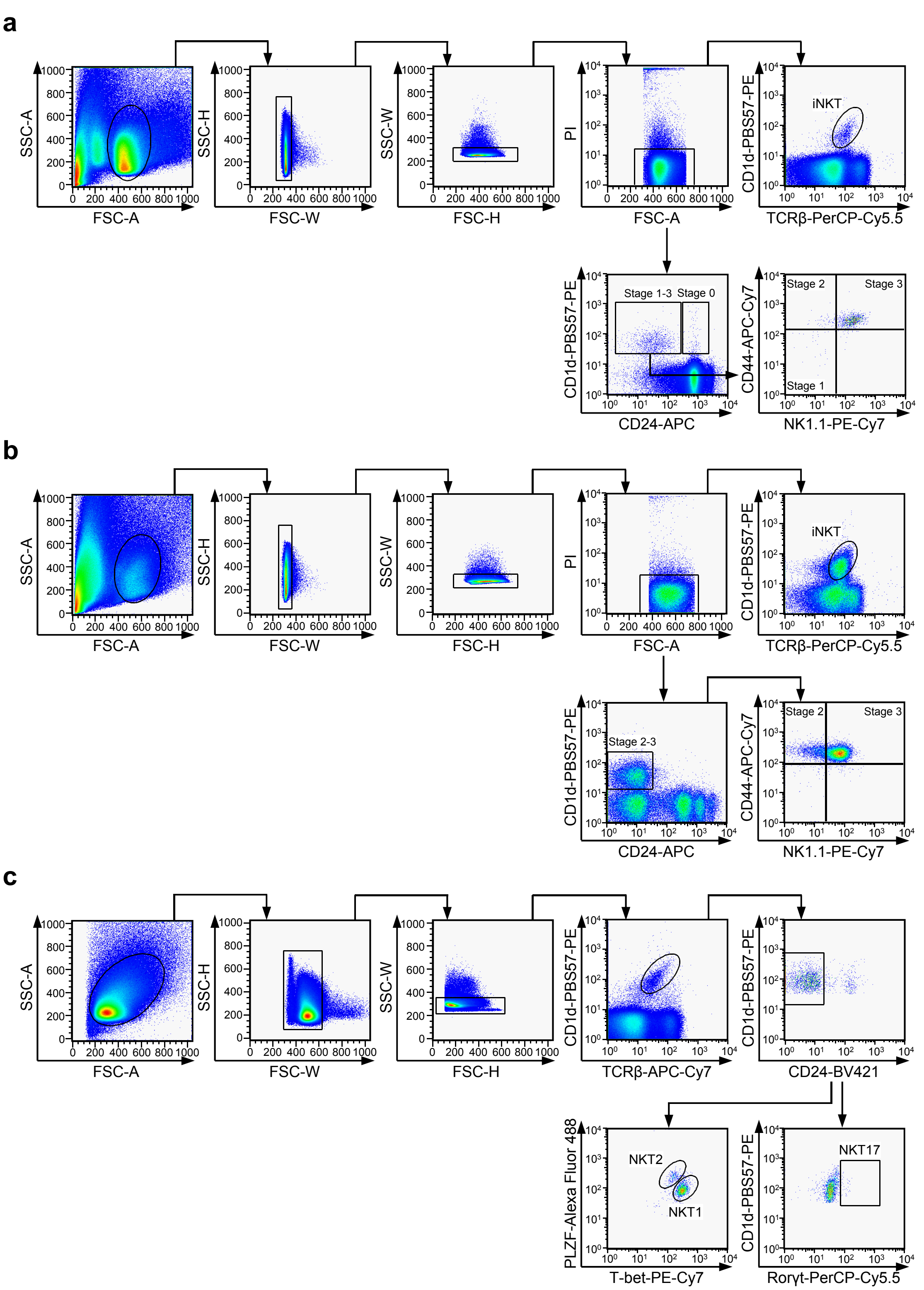

Supplementary Figure 1. Gating strategies for flow-cytometric analysis of iNKT cells. (a) Gating strategy to analyze the total iNKT cells (TCRβ^{int}CD1d-PBS57^{+}), stage 0 iNKT cells (CD24^{high}CD1d-PBS57^{+}), stage 1-3 iNKT cells (CD24^{low}CD1d-PBS57^{+}), stage 1 iNKT cells (CD24^{low}CD1d-PBS57^{+}CD44^{-}NK1.1^{-}), stage 2 iNKT cells (CD24^{low}CD1d-PBS57^{+}CD44^{+}NK1.1^{-}) and stage 3 iNKT cells (CD24^{low}CD1d-PBS57^{+}CD44^{+}NK1.1^{+}) in the thymi presented on Fig. 1a,c and d. (b) Gating strategy to analyze the total iNKT cells (TCRβ^{int}CD1d-PBS57^{+}) in the spleens, livers and lungs presented on Fig. 1a and Fig. 7d, stage 2-3 iNKT cells (CD24^{low}CD1d-PBS57^{+}), stage 2 iNKT cells (CD24^{low}CD1d-PBS57^{+}CD44^{+} NK1.1^{-}) and stage 3 iNKT cells (CD24^{low}CD1d-PBS57^{+}CD44^{+}NK1.1^{+}) in the spleens and livers presented on Fig. 1c and d. (c) Gating strategy to analyze the NKT1 cells (CD24^{low}TCRβ^{int}CD1d-PBS57^{+}PLZF^{low}T-bet^{high}), NKT2 cells (CD24^{low}TCRβ^{int}CD1d-PBS57^{+} PLZF^{high}T-bet^{low}) and NKT17 cells (CD24^{low}TCRβ^{int}CD1d-PBS57^{+}RORγt^{high}) in the thymi, spleens and livers presented on Fig. 2a.

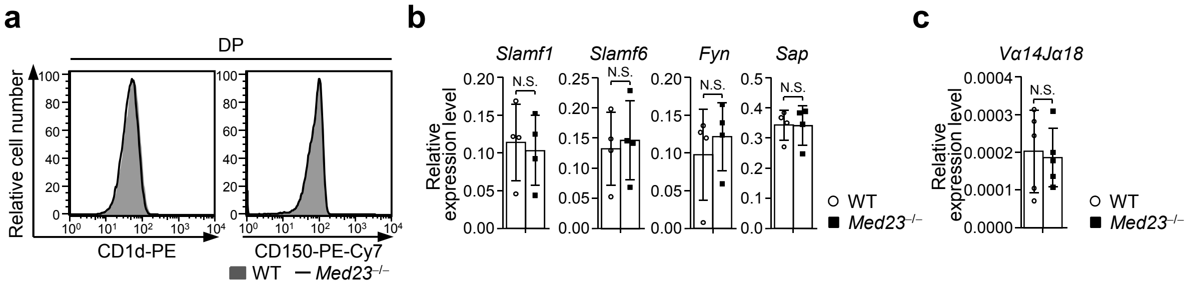

Supplementary Figure 2. Med23 deficiency does not affect Va14Ja18 rearrangement and the expression of key signaling molecules in iNKT cell precursors. (a) Representative histograms of surface CD1d and CD150 (Slamf1) in DP cells from WT and *Med23*$^{-/-}$ mice. (b) The transcriptional levels of *Slamf1*, *Slamf6*, *Fyn* and *Sap* in sorted DP cells from WT and *Med23*$^{-/-}$ mice (n = 4). (c) Quantitative RT-PCR analysis of *Vα14Jα18* TCR transcript in sorted DP cells from WT and *Med23*$^{-/-}$ mice (n = 5). All expression levels were normalized to *Gapdh* expression.

The data are presented as the mean ± s.d. For all panels: N.S.: no significance. All data are representative of or combined from at least three independent experiments.

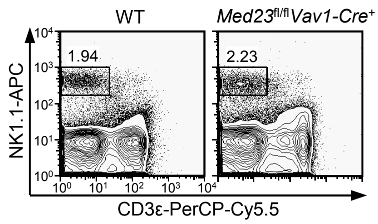

Supplementary Figure 3. Med23 does not regulate NK1.1 expression directly. Flow cytometric analysis of NK cells in the spleens from WT and *Med23*$^{fl/fl}$*Vav1-Cre*$^{+}$ mice.

The data are representative of at least three independent experiments.

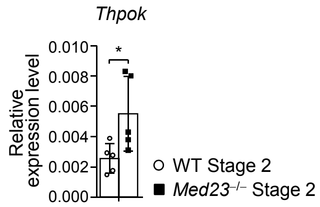

Supplementary Figure 4. Loss of Med23 upregulates ThPOK expression in stage 2 iNKT cells. Quantitative RT-PCR analysis of *Thpok* mRNA levels in sorted stage 2 iNKT cells in the thymi from WT and *Med23*$^{-/-}$ mice ($n = 5$). The expression levels were normalized to *Gapdh* expression.
The data are presented as the mean ± s.d. For the panel: *$P < 0.05$ by Student's *t*-test. The data are combined from at least three independent experiments.

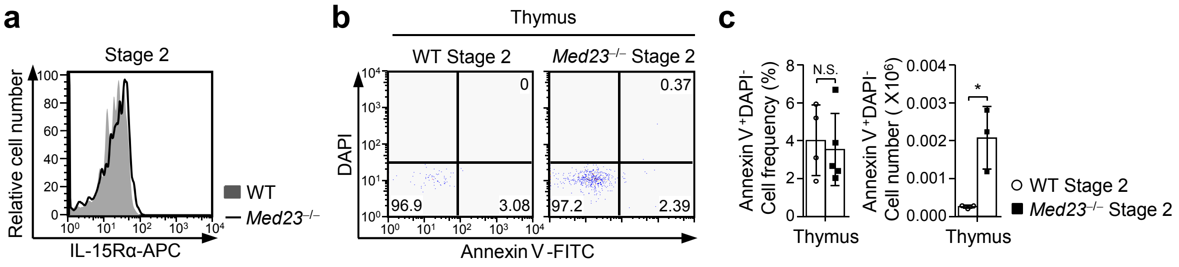

Supplementary Figure 5. Med23 deficiency does not affect the expression of IL-15 receptor and survival of stage 2 iNKT cells. (a) Representative histograms of surface IL-15Rα in thymic stage 2 iNKT cells from WT and *Med23*$^{-/-}$ mice. (b) Survival of stage 2 iNKT cells from WT and *Med23*$^{-/-}$ mice was assessed by flow cytometric analysis of Annexin Ⅴ and DAPI staining. (c) The frequency and cell number of Annexin Ⅴ$^{+}$DAPI^{-} stage 2 iNKT cells from WT and *Med23*$^{-/-}$ mice (Annexin Ⅴ$^{+}$DAPI^{-} Cell frequency, WT, $n = 4$, KO, $n = 5$; Annexin Ⅴ$^{+}$DAPI^{-} Cell number, $n = 3$).
The data are presented as the mean ± s.d. For all panels: *$P < 0.05$ by Student's *t*-test; N.S.: no significance. All data are representative of or combined from at least three independent experiments.

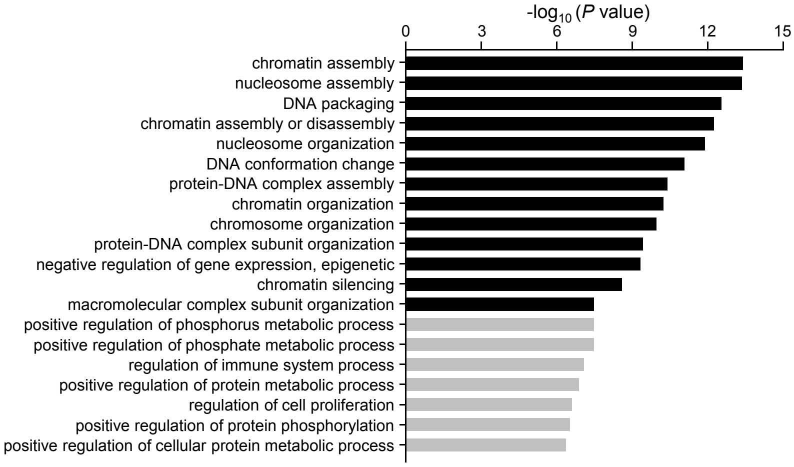

Supplementary Figure 6. Gene expression analysis of WT and *Med23*$^{-/-}$ stage 2 iNKT cells. Pathway enrichment analysis of differentially expressed genes (modified Fisher's exact *p*-value < 0.05) in thymic stage 2 iNKT cells from WT and *Med23*$^{-/-}$ mice (black: categories related to chromosome assembly).

The data are combined from at least three independent experiments.

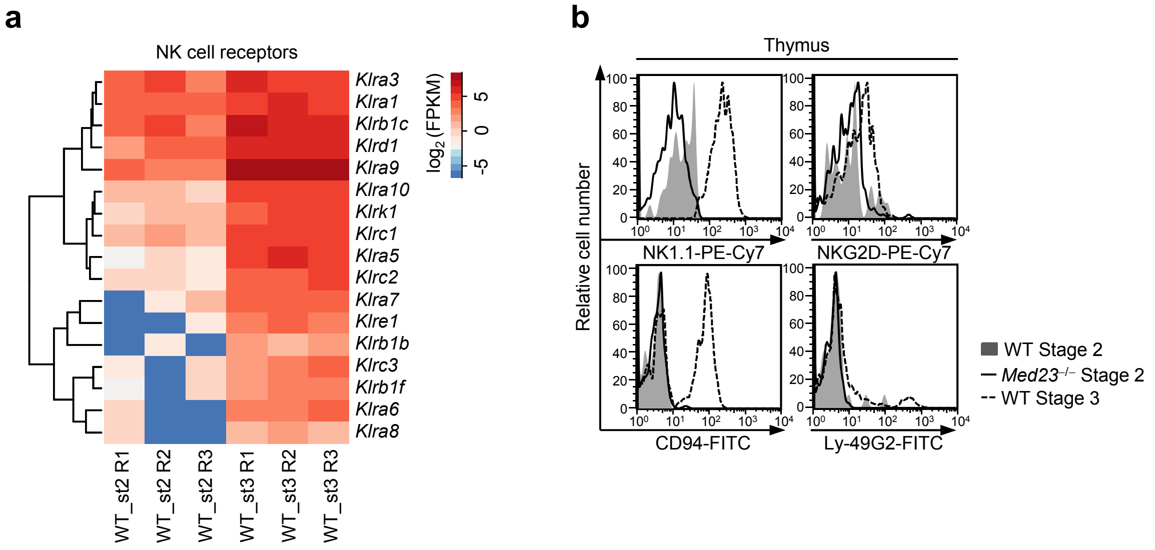

Supplementary Figure 7. Med23 deficiency abolishes NK cell characteristics of iNKT cells. (a) The expression of NK cell receptors in WT stage 2 and stage 3 cells. (b) Representative histograms of surface NK1.1, NKG2D, CD94 and Ly-49G2 in thymic WT stage 2 and stage 3 cells and *Med23*$^{-/-}$ stage 2 cells.

The data are representative of or combined from at least three independent experiments.

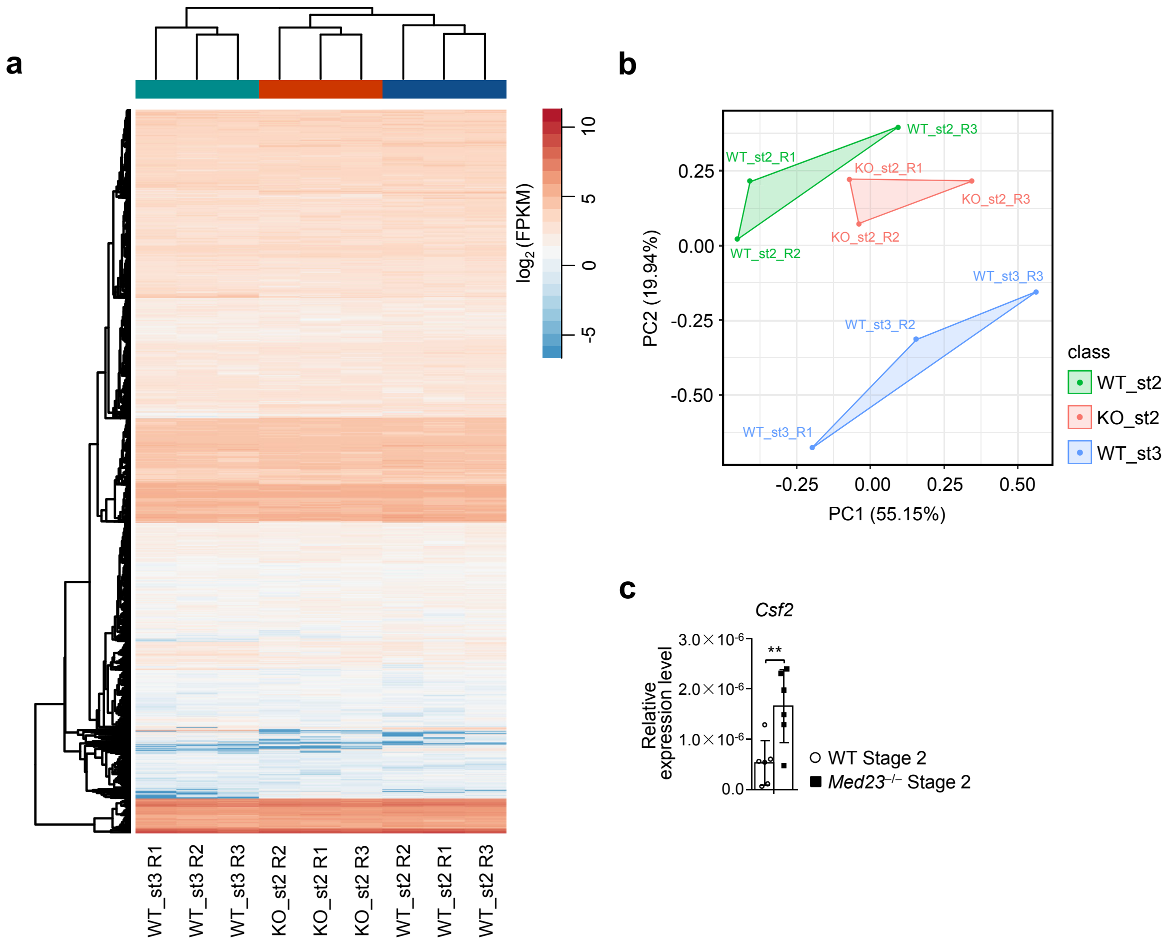

Supplementary Figure 8. Gene expression analysis and Csf-2 expression of *Med23*$^{-/-}$ stage 2 iNKT cells. (a) RNA-seq analysis of WT stage 2 and stage 3 cells and *Med23*$^{-/-}$ stage 2 cells in the thymi. (b) Principle component analysis of thymic stage 2 and stage 3 cells from WT mice and stage 2 cells from *Med23*$^{-/-}$ mice. (c) Quantitative RT-PCR analysis of *Csf2* transcript in sorted stage 2 iNKT cells of the thymus from WT and *Med23*$^{-/-}$ mice (n = 6). The expression levels were normalized to *Gapdh* expression.

The data are presented as the mean ± s.d. For all panels: $^{**}P < 0.01$ by Student's t-test. All data are combined from at least three independent experiments.

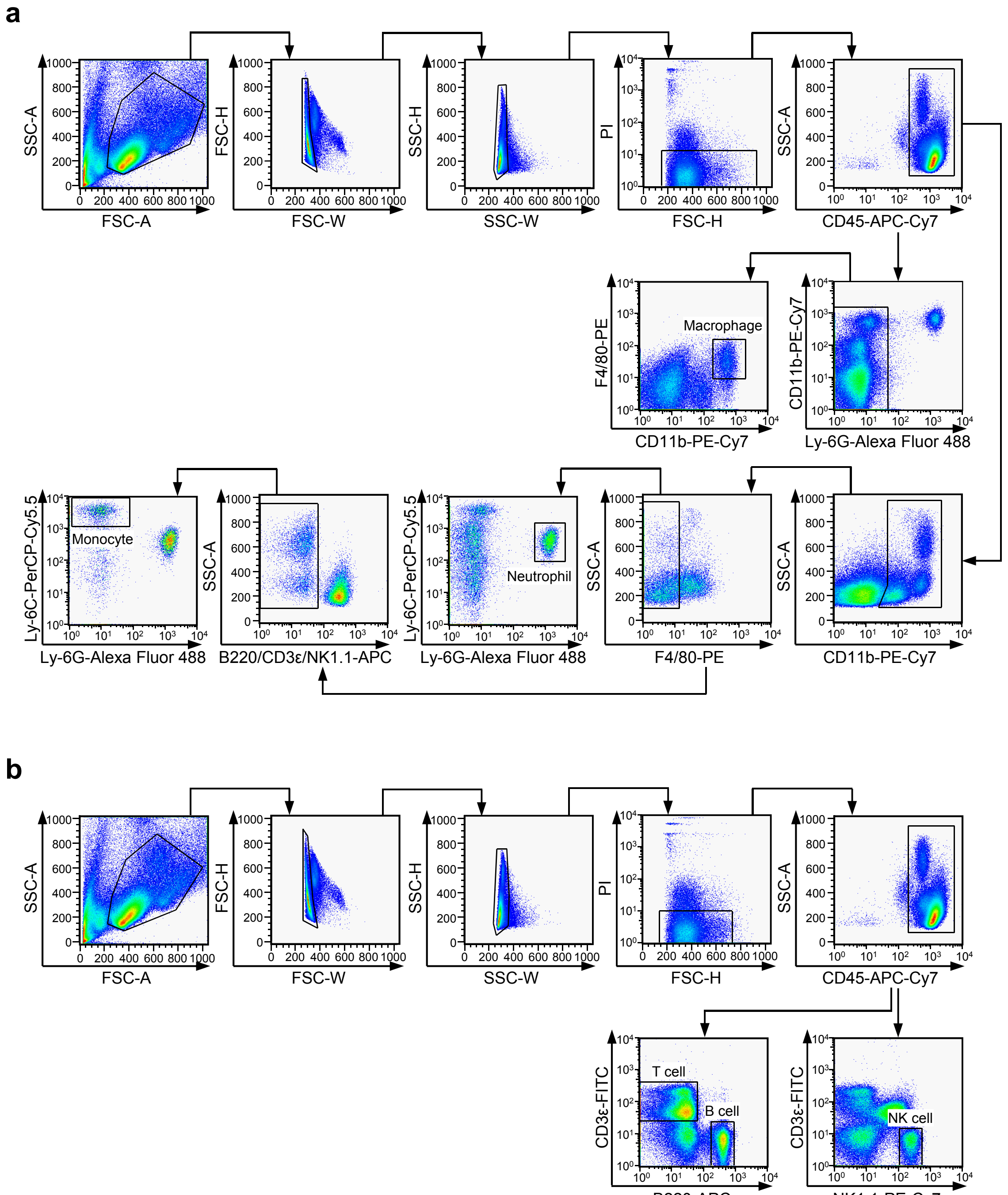

Supplementary Figure 9. Gating strategies for flow-cytometric analysis of leukocytes. (a) Gating strategy to analyze the neutrophils (CD45^{+}CD11b^{+}F4/80^{-}Ly-6G^{+}Ly-6C^{+}), monocytes (CD45^{+}CD11b^{+}F4/80^{-}CD3ε^{-}B220^{-}NK1.1^{-}Ly-6G^{-}Ly-6C^{+}) and macrophages (CD45^{+}Ly-6G^{-}CD11b^{+}F4/80^{+}) in the livers presented on Fig. 6a. (b) Gating strategy to analyze T cells (CD45^{+}B220^{-}CD3ε^{+}), B cells (CD45^{+}CD3ε^{-}B220^{+}), and NK cells (CD45^{+} CD3ε^{-}NK1.1^{+}) in the livers presented on Fig. 6a.

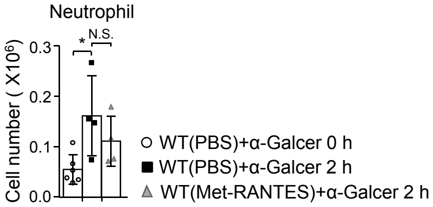

Supplementary Figure 10. Recruitment capacity of iNKT cells is slightly reduced by treatment with the CCL5 antagonist Met-RANTES. WT mice were intraperitoneally injected PBS or Met-RANTES 30 min before α-GalCer administration. The absolute number of neutrophils in the livers of WT mice were measured 0 h or 2 h after α-GalCer stimulation (0 h, n = 6; 2 h, n = 4).

The data are presented as the mean ± s.d. For the panel: *$P < 0.05$ by Student's t-test; N.S.: no significance. The data are combined from at least three independent experiments.

Supplementary Table 1. Primer sequences for genotyping and qPCR

Primer name	Sequence	Application
Med23 forward	5′ -GCGGCCGCTATATGCACTGTTAGTGATT-3′	genotyping
Med23 reverse	5′ -GTCGACCTTAGAAGAAAGCTCAAACAT-3′	genotyping
Cd4-Cre forward	5′ -CCTGATCCTGGCAATTTCGG -3′	genotyping
Cd4-Cre reverse	5′ -CCCAACCAACAAGAGCTC-3′	genotyping
Vav1-Cre forward	5′ -AGATGCCAGGACATCAGGAACCTG-3′	genotyping
Vav1-Cre reverse	5′ -ATCAGCCACACCAGACACAGAGATC-3′	genotyping
Gapdh forward	5′ -CGACTTCAACAGCAACTCCCACTCTTCC-3′	qPCR
Gapdh reverse	5′ -TGGGTGGTCCAGGGTTTCTTACTCCTT-3′	qPCR
Slamf1 forward	5′ -TAATCTTCATCCTGGTTTTCACGGC-3′	qPCR
Slamf1 reverse	5′ -CCCTGATTTCTGTACTTGGGCATA-3′	qPCR
Slamf6 forward	5′ -ACTCCGCCTGTCAGAGGATGGT-3′	qPCR
Slamf6 reverse	5′ -CTAGAACGCCATTCATTAGCTGGG-3′	qPCR
Fyn forward	5′ -GCGTGACCTCCATCCCGAACTA-3′	qPCR
Fyn reverse	5′ -TAAAGCGCCACAAACAGTGTCAC-3′	qPCR
Sap forward	5′ -AGCGAGAGTGTCCCTGGCGT-3′	qPCR
Sap reverse	5′ -GATCCGGCTTCTGAAACGCTG-3′	qPCR
Vα14Jα18 forward	5′ -GGATGACACTGCCACCTACA-3′	qPCR
Vα14Jα18 reverse	5′ -CTGAGTCCCAGCTCCAAAA-3′	qPCR
Med23 forward	5′ -GCCTATTGCCGGCCTACTTT-3′	qPCR
Med23 reverse	5′ -CTGTGGGCCTGAAGGTATCC-3′	qPCR
Tbx21 forward	5′ -CAACAACCCCTTTGCCAAAG-3′	qPCR
Tbx21 reverse	5′ -TCCCCCAAGCAGTTGACAGT-3′	qPCR
Egr2 forward	5′ -CCTTTGACCAGATGAACGGAGTG-3′	qPCR
Egr2 reverse	5′ -CTGGTTTCTAGGTGCAGAGATGG-3′	qPCR
Med1 forward	5′ -CTTGTGCGTCAAGTAATGGAGA-3′	qPCR
Med1 reverse	5′ -TTCCAAACGATCAGTCATTGCT-3′	qPCR
Tsc-1 forward	5′ -AAGCATCCTGACACCACCAAG-3′	qPCR
Tsc-1 reverse	5′ -GGATCTCCAGTTCCAACACCC-3′	qPCR
Pten forward	5′ -TGGATTCGACTTAGACTTGACCT-3′	qPCR
Pten reverse	5′ -GCGGTGTCATAATGTCTCTCAG-3′	qPCR
c-Jun forward	5′ -GTCCCCTATCGACATGGAGTCT-3′	qPCR
c-Jun reverse	5′ -GGAGTTTTGCGCTTTCAAGGT-3′	qPCR
Batf forward	5′ -CACAGAAAGCCGACACCCTT-3′	qPCR
Batf reverse	5′ -GCTGTTTGATCTCTTTGCGGA-3′	qPCR
Xcl1 forward	5′ -GCGATCAAGACTGTGGAT-3′	qPCR
Xcl1 reverse	5′ -CTGCTTAACGAGTGAGGAA-3′	qPCR
Ccl5 forward	5′ -TTTGCCTACCTCTCCCTCG-3′	qPCR
Ccl5 reverse	5′ -CGACTGCAAGATTGGAGCACT-3′	qPCR
Ccl4 forward	5′ -TTCCTGCTGTTTCTCTTACACCT-3′	qPCR
Ccl4 reverse	5′ -CTGTCTGCCTCTTTTGGTCAG-3′	qPCR
Thpok forward	5′ -AGTCTGTCACAAGATAATCCA-3′	qPCR
Thpok reverse	5′ -GGTTCTTGAGGTCGTAGC-3′	qPCR
Csf2 forward	5′ -CTCTAACGAGTTCTCCTTCA-3′	qPCR
Csf2 reverse	5′ -GGCAGTATGTCTGGTAGTAG-3′	qPCR

Supplementary Table 2. Fluorescently conjugated protein or antibodies used for flow cytometry

Target	Fluorochrome	Clone	Manufacturer	Catalog	Dilution ratio (vol/vol)
Cell surface stain					
TCRβ	FITC	H57-597	BD	553171	1:150
TCRβ	PerCP-Cy5.5	H57-597	BD	560657	1:150
TCRβ	APC-Cy7	H57-597	BioLegend	109220	1:150
CD11b	PE-Cy7	M1/70	BD	552850	1:150
CD1d	PE	1B1	BD	553846	1:150
CD3ε	APC	145-2C11	BD	553066	1:150
CD3ε	FITC	145-2C11	BioLegend	100306	1:150
CD3ε	PerCP-Cy5.5	145-2C11	BD	551163	1:150
CD45	APC-Cy7	30-F11	BD	561037	1:150
CD94	FITC	18d3	eBioscience	11-0941-81	1:150
Ly-49G2	FITC	eBio4D11	eBioscience	11-5781-81	1:150
F4/80	PE	BM8	eBioscience	12-4801-80	1:150
NKG2D	PE-Cy7	CX5	eBioscience	25-5882-81	1:150
CD24	FITC	M1/69	BioLegend	101806	1:150
CD24	APC	M1/69	BioLegend	101814	1:150
CD24	BV421	M1/69	BioLegend	101826	1:150
Ly-6G	AF 488	1A8	BioLegend	127625	1:300
Ly-6C	PerCP-Cy5.5	HK1.4	BioLegend	128011	1:300
NK1.1	PE-Cy7	PK136	BioLegend	108714	1:150
NK1.1	APC	PK136	BioLegend	108710	1:150
B220	APC	RA3-6B2	BioLegend	103211	1:150
CD44	APC-Cy7	IM7	BioLegend	103028	1:150
CD44	FITC	IM7	BD	553133	1:150
CD150	PE-Cy7	TC15-12F12.2	BioLegend	115913	1:150
IL-15Ra	APC	DNT15Ra	eBioscience	17-7149-82	1:150
Annexin V	FITC		BioLegend	640945	1:20
Intracellular stain					
RORγt	PerCP-Cy5.5	Q31-378	BD	562683	1:12
RORγt	PE	B2D	eBioscience	12-6981-80	1:30
PLZF	AF 488	Mags.21F7	eBioscience	53-9320-82	1:15
IFN-γ	AF 488	XMG1.2	eBioscience	53-7311-82	1:150
CCL5	PE	2E9/CCL5	BioLegend	149103	1:150
T-bet	PE	4B10	BioLegend	644809	1:12
T-bet	PE-Cy7	4B10	BioLegend	644823	1:30
IL-4	PE-Cy7	11B11	BioLegend	504118	1:150

AF, Alexa Fluor; APC, allophycocyanin; BV, brilliant violet; FITC, fluorescein isothiocyanate; PE, phycoerythrin; PerCP, peridinin–chlorophyll–protein.
